# Imaging gravity-induced lung water redistribution with automated inline processing at 0.55 T cardiovascular magnetic resonance

**DOI:** 10.1186/s12968-022-00862-4

**Published:** 2022-06-06

**Authors:** Felicia Seemann, Ahsan Javed, Rachel Chae, Rajiv Ramasawmy, Kendall O’Brien, Scott Baute, Hui Xue, Robert J. Lederman, Adrienne E. Campbell-Washburn

**Affiliations:** Cardiovascular Branch, Division of Intramural Research, National Heart, Lung, and Blood Institute, National Institutes of Health, Bethesda, MD 20892 USA

**Keywords:** Cardiovascular magnetic resonance, Lung water, Lung imaging, Heart failure, Low-field MRI

## Abstract

**Background:**

Quantitative assessment of dynamic lung water accumulation is of interest to unmask latent heart failure. We develop and validate a free-breathing 3D ultrashort echo time (UTE) sequence with automated inline image processing to image changes in lung water density (LWD) using high-performance 0.55 T cardiovascular magnetic resonance (CMR).

**Methods:**

Quantitative lung water CMR was performed on 15 healthy subjects using free-breathing 3D stack-of-spirals proton density weighted UTE at 0.55 T. Inline image reconstruction and automated image processing was performed using the Gadgetron framework. A gravity-induced redistribution of LWD was provoked by sequentially acquiring images in the supine, prone, and again supine position. Quantitative validation was performed in a phantom array of vials containing mixtures of water and deuterium oxide.

**Results:**

The phantom experiment validated the capability of the sequence in quantifying water density (bias ± SD 4.3 ± 4.8%, intraclass correlation coefficient, ICC = 0.97). The average global LWD was comparable between imaging positions (supine 24.7 ± 3.4%, prone 22.7 ± 3.1%, second supine 25.3 ± 3.6%), with small differences between imaging phases (first supine vs prone 2.0%, p < 0.001; first supine vs second supine − 0.6%, p = 0.001; prone vs second supine − 2.7%, p < 0.001). In vivo test–retest repeatability in LWD was excellent (− 0.17 ± 0.91%, ICC = 0.97). A regional LWD redistribution was observed in all subjects when repositioning, with a predominant posterior LWD accumulation when supine, and anterior accumulation when prone (difference in anterior–posterior LWD: supine − 11.6 ± 2.7%, prone 5.5 ± 2.7%, second supine − 11.4 ± 2.9%). Global LWD maps were calculated inline within 23.2 ± 0.3 s following the image reconstruction using the automated pipeline.

**Conclusions:**

Redistribution of LWD due to gravitational forces can be depicted and quantified using a validated free-breathing 3D proton density weighted UTE sequence and inline automated image processing pipeline on a high-performance 0.55 T CMR system.

## Background

Pulmonary edema, or lung water, is the accumulation of fluid that has leaked from the vasculature into the pulmonary interstitial space or alveoli, which may cause dyspnea and exercise intolerance [1, 2]. Exercise intolerance due to lung water is a key feature in at least 50% of heart failure patients [3, 4], often manifesting as a dynamic process at early stages of the disease with left ventricular filling pressures and lung water densities that are normal at rest but increased during physical activity [5]. Furthermore, lung water is predictive of cardiac events in patients at risk of developing or with confirmed heart failure [6–8]. Quantification and dynamic assessments of lung water density (LWD) are therefore valuable for evaluation of this patient cohort.

Clinically available tests to assess LWD include chest X-ray, computed tomography (CT), ultrasound, and heart catheterizations [9–11]. These exams are invasive or expose patients to ionizing radiation, and in addition, are only qualitative or semi-quantitative. Recent studies have proposed cardiovascular magnetic resonance (CMR) as a promising noninvasive, ionizing radiation-free, and quantitative method to measure LWD, using half-Fourier single-shot turbo spin-echo (HASTE) or ultrashort echo time (UTE) sequences at 1.5 T or 3 T [6, 12, 13]. These methods, however, rely on offline image processing using either observer-dependent manual segmentations or automated but time-consuming region-growing algorithms, and their capability to image dynamic changes in lung water has not yet been studied.

In this study, we develop and validate a method to image lung water using a 3D free-breathing UTE sequence on a high-performance low field strength 0.55 T CMR system [14]. High-performance 0.55 T CMR is well suited for structural lung imaging due to the improved field homogeneity resulting in reduced artifacts from susceptibility gradients, and thus improved parenchymal imaging [15–18]. We also implement a fast and fully automated neural network-based inline image processing pipeline, allowing LWD maps to be displayed directly on the CMR scanner interface. Water density quantification is validated using a phantom. As a precursor to assessment of dynamic lung water measurements during exercise stress, we aimed to assess a regional redistribution of lung water and therefore deployed our method to image a gravity-induced redistribution of lung water in healthy subjects in supine and prone positions.

## Methods

### Study population

Imaging was approved by the local Institutional Review Board (ClinicalTrails.gov identifier NCT03331380) with written informed consent obtained from all participants. We prospectively performed research CMR examinations in 15 healthy subjects (29 ± 7 years, 6 women).

### Imaging protocol

Imaging was performed on a prototype high-performance 0.55 T CMR system (prototype MAGNETOM Aera, Siemens Healthineers, Erlangen, Germany) using phased-array receiver coils retuned for 0.55 T, including an 18-channel spine array and a 6-channel body coil [15]. LWD was measured with a modified version of a previously described 3D free-breathing proton density weighted stack-of-spirals golden-angle UTE spoiled-gradient echo pulse sequence [14]. Typical imaging parameters were TE/TR/θ = 0.56 ms/9 ms/1°, stack-of-spirals with 171 interleaves, spiral readout duration 5.0 ms, field of view 450 × 450 × 252 mm, 3.5 mm isotropic resolution, 72–80 coronal slices covering both lungs, and slice oversampling factor 11.1%. Acquisition time was ~ 2.5 min, but varied slightly with anterior–posterior field-of-view (i.e. number of slices). Superior-inferior navigator readouts were acquired every 144 ms. The navigator was used to extract the respiratory signal, which was used to bin and reconstruct 40% of the data of the most stable respiratory phase. Image reconstruction was performed in the Gadgetron reconstruction framework using the pipeline described in Javed et al. [14].

**Fig. 1:**
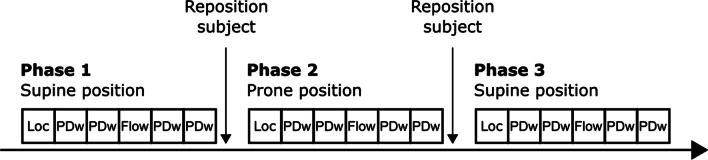
Schematic illustration of the imaging protocol, divided into three phases. Each phase included acquisition of localizers (Loc), two proton density weighted (PDw) images, one main pulmonary artery flow, followed by two additional proton density weighted images. Subjects were in the supine position in the first phase, in the prone position for the second phase, and back into a supine position for the third phase. Repositioning of the subject, coils, running calibrations and localizers took approximately 6 min

A gravity-induced redistribution of lung water was achieved by repositioning subjects between the supine and prone position. The study protocol consisted of three phases (Fig. 1), In the first phase, imaging was performed in a supine position, the second in a prone position, and in the third phase imaging was repeated in a supine position. The subject was removed from the magnet bore for repositioning and coil placement. In each phase, shimming, localizers and four 3D UTE lung water images were acquired.

To control for potential variations in pulmonary blood flow between positions, main pulmonary artery flow was measured using a free-breathing phase contrast gradient echo sequence with pulse triggering. Typical imaging parameters were TE/TR/θ = 4.19 ms/27.44 ms/30°, VENC 200 cm/s, field of view 400 × 400 mm, resolution 2.1 × 2.1 × 6 mm, bandwidth = 299 Hz/Px. Flow images were not acquired in one subject for technical reasons. Cardiac output was quantified from the flow images using Segment, by semi-automatically delineating the main pulmonary artery over time [19]. This analysis was not part of the inline image processing pipeline.

### Phantom validation

Quantitative accuracy and test–retest validation of the proton density weighted sequence were performed using a custom phantom of 10 vials containing a 50 ml mixture of distilled water and deuterium oxide, which were immersed in a water filled container. Water concentrations ranged between 10 and 100% in increments of 10%. Deuterium oxide provides no CMR signal, meaning that the proton densities measured should be directly proportional to the water content in each vial. The phantom was imaged three times on different days within 2 weeks, using the same sequence and imaging parameters as for the study participants.

**Fig. 2:**
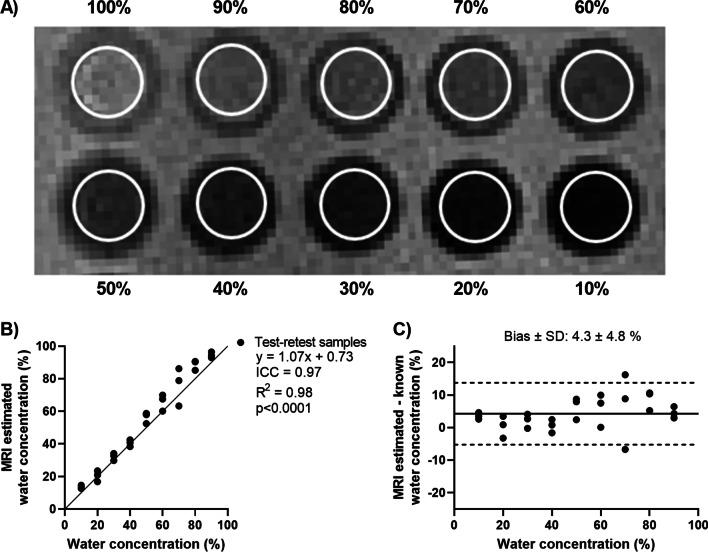
Quantitative phantom validation in array of vials with varying water and deuterium oxide concentrations. **A** CMR image with analyzed regions of interests (white circles). **B** Correlation plot between known water concentrations used to make the phantom and CMR-estimated water concentrations relative the 100% water vial, showing a linear relation. **C** Bland–Altman plot, showing a low bias between the metrics. Three test–retest measurements were made on different days. *ICC* intraclass correlation coefficient

Phantom images were analyzed using the software Segment (Medviso AB, Lund, Sweden) [20]. Regions of interest contouring each vial were placed in a central slice, to avoid partial volume effects (Fig. 2A). Water densities were calculated as the ratio of the average signal intensities in each vial and the average signal intensity in the vial containing 100% water. Calculated and known water densities from all phantom experiments were compared.

### Lung segmentation

**Fig. 3:**
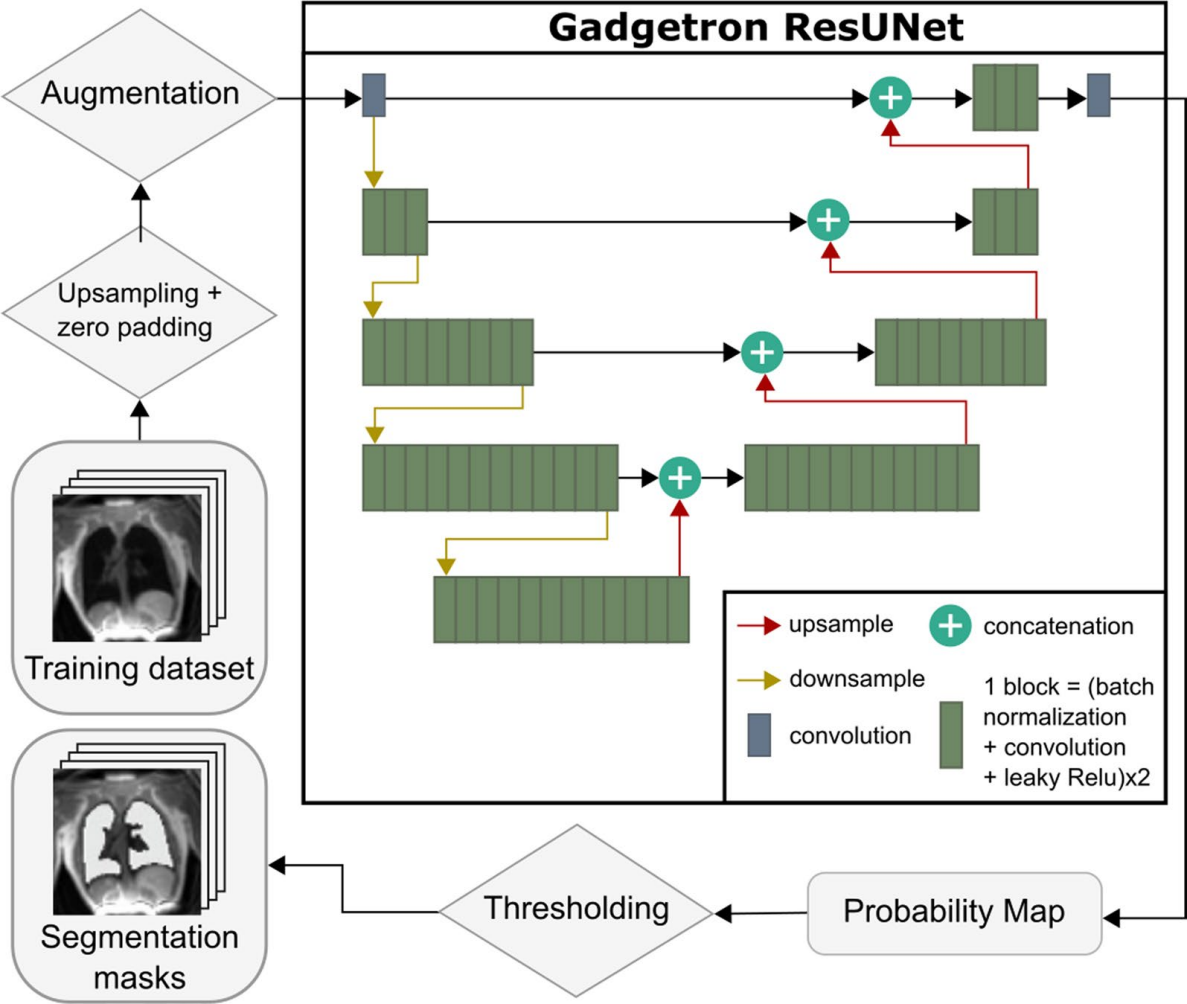
Diagram of the training workflow and the 2D residual U-Net architecture. Data was augmented to improve generalization of the network. Input image slices pass through three downsampling layers followed by a bridge layer and four upsampling layers. Each block in the Gadgetron U-Net diagram represents two cycles of batch normalization, convolution layer, and leaky ReLU activation. The final layer was a 1 × 1 convolution used to convert the output channels into a probability map, and no nonlinearity function was added after the output convolution. The U-Net outputs a probability map, which was then thresholded to create 2D lung segmentation masks

Automated lung segmentation was performed using a trained convolutional neural network which was specifically trained for UTE lung images acquired at 0.55 T. The network had a residual U-net semantic segmentation architecture which have previously been used successfully in biomedical imaging applications, offering improved performance for deeper networks with fewer parameters and overcoming degradation problems [21–25], and was developed in PyTorch based on the Gadgetron AI for CMR Imaging repository (Fig. 3) [23, 26].

Model training was performed using lung images from 114 human subjects and 23 swine, acquired with various T1 and proton density weighted (flip angle 20° and 1°, respectively) stack-of-spirals ultrashort echo time sequences using the high-performance 0.55 T CMR system. Lung segmentation was performed semi-automatically with an active contour algorithm followed by manual corrections, avoiding major vasculature and airways [2]. A total of 467 3D images (~ 16 k 2D image slices) from healthy subjects (n = 40), lymphangioleiomyomatosis patients (n = 74), and swine (n = 23) were used in both coronal and sagittal slices with varying spatial resolution. The dataset was randomly divided into training and validation sets, where 80% of image-mask pairs were used for training and the remaining 20% were used for validation. Furthermore, a separate test set of 761 3D images (~ 43,500 2D slices) from 31 healthy subjects and 9 swine was prepared to evaluate the segmentation model. The data included for lung water measurements in this study was not included in the training. Imaging used for U-Net training was approved by the local Institutional Review Board and Institutional Animal Care and Use Committee, with written informed consent obtained from all human participants.

The network was trained with 2D image slices as input. Images and lung segmentation masks were up-sampled to achieve a consistent pixel resolution between 1 and 1.5 mm^2^ for all images. Data augmentation was performed by adding random permutations, blurring, scaling, rotating, and noise to improve generalization. During training, the performance of the network was evaluated through validation loss calculated with Soft-Jaccard loss function and the Dice coefficient. A hyperparameter sweep was performed to determine the batch size (32 or 64), the number of up-sampling and down-sampling layers (2, 3 or 4), and the number of block computations for each layer (2, 3, 4, 6, 8, or 12) that resulted in the lowest validation loss. One block computation consists of two cycles of batch normalization, convolution, and leaky ReLU activation. Model performance was evaluated in the test set using the Dice coefficient and quantified lung volume.

### Inline image analysis

**Fig. 4:**
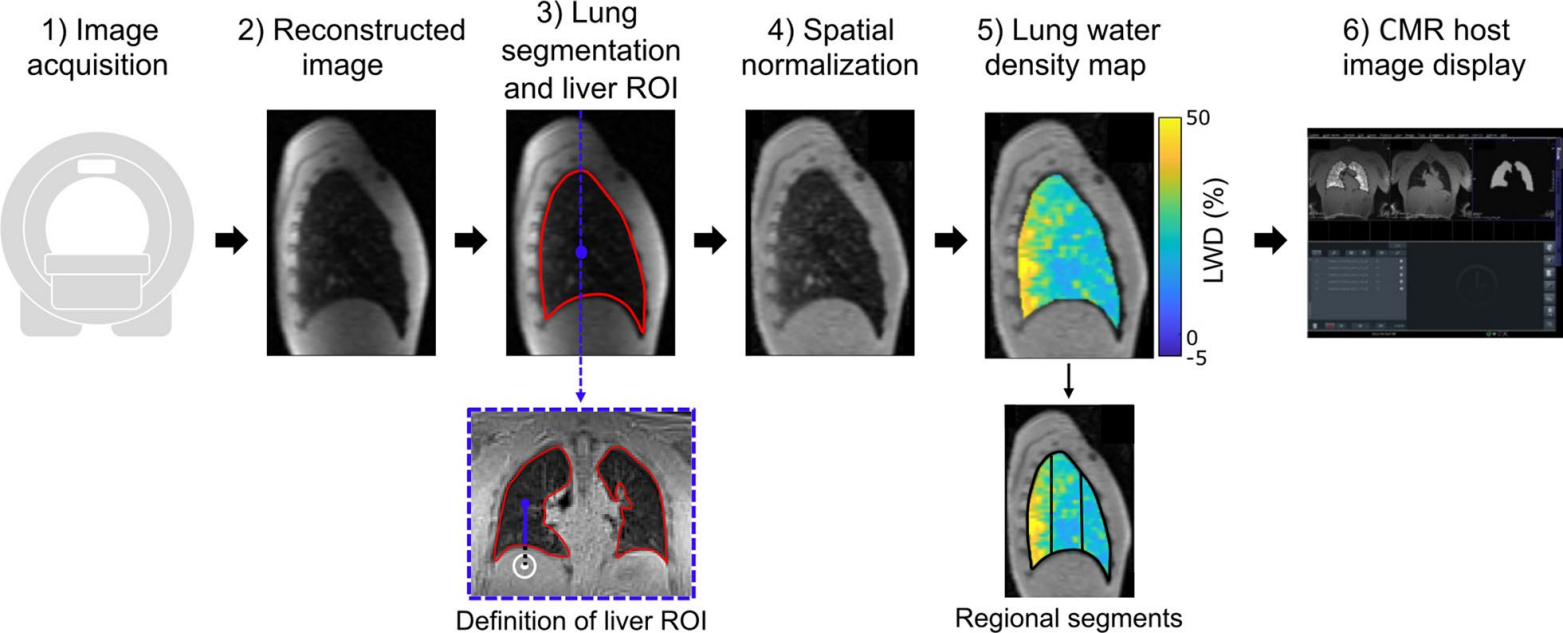
The automated image processing pipeline. **1**, **2** The acquired image was reconstructed inline using the Gadgetron image reconstruction framework. **3** Lung segmentation (red) was performed using a trained U-Net. A region-of-interest (ROI) in the liver was automatically placed under the right lung (white circle) for quantitative purposes. The positioning of the liver ROI was determined from the centroid of the right lung segmentation (blue circle), with the liver ROI was placed in the coronal slice passing through the centroid (blue dashed line). The positioning of the liver ROI was placed 8.75 mm below the bottom of the in-plane lung segmentation (black dashed line) in the centroid column (blue line). **4** Sample of a coil spatially normalized resliced sagittal slice of a healthy subject in the supine position. **5** Quantitative pixel-wise lung water density (LWD) maps relative 70% of the signal intensity in the liver were derived. To enable regional analysis, anterior, mid, and posterior segments were defined (black), that were derived by assigning each segment an equal number of coronal slices. **6** Reconstructed images were streamed back and displayed to CMR host, including a LWD map overlayed on the acquired image

A fully automated image processing pipeline that derives a 3D pixel-wise LWD map was implemented in Matlab (Mathworks, Natick, Massachusetts, USA) and is illustrated in Fig. 4.

First, lung segmentation was performed using the trained neural network and a circular 12.5 cm^2^ region-of-interest (ROI) was automatically placed in the liver. The liver ROI was placed in the slice pertaining to the centroid of the right lung segmentation. The in-plane positioning was defined through the center point of the circle, which was placed 8.75 mm below the bottom of the right lung, in the column of the centroid of the segmented right lung in 3D (Fig. 4).

Second, spatial normalization to avoid surface coil shading was performed by dividing the image by a normalization map that was fitted to the signal intensities in the body, as previously proposed by Meadus et al. [12]. Signal intensities pertaining to the body were defined by thresholding the image to the average signal intensity in the imaged 3D volume, and subsequently removing the segmented lung tissue. The normalization map was fitted and interpolated over the body and lungs slice-by-slice in the coronal orientation, using least square linear regression with Tikhonov regularization and an L-curve method derived smoothness parameter [12, 27, 28].

Third, pixel-wise 3D LWD maps were calculated as the ratio of lung tissue signal intensities and the average liver signal intensity, with the assumption that the hepatic water density is 70% [6, 29]. The average global LWD were reported for all subject positions and sequence repetitions. Global LWD derived with the fully automated pipeline were compared to results using manually performed lung segmentations and placement of the liver ROI.

The image processing pipeline was implemented in Matlab (Mathworks) and integrated with the Gadgetron framework using Gadgetron’s external interface, allowing inline processing on the CMR scanner. Image reconstruction and analysis were performed on a dual-socket Xeon E5-2600 computer with 512 GB RAM and 3x NVIDIA QUADRO RT X 8000 s (48 GB vRAM). To enable further offline analysis and potential manual corrections, the pipeline was also implemented as a plugin to the medical image analysis software Segment v3.2 which is freely available for research purposes [20]. Both implementations are available open source (https://github.com/NHLBI-MR/lung_water_pipeline).

### Lung water dynamics

The gravity-induced redistribution of lung water was visualized in LWD maps derived from images in the supine and prone positions. The isotropic 3D volumes were resliced into the sagittal view for assessment of the anterior–posterior LWD gradient. Regional changes of lung water distribution in the anterior–posterior direction were assessed by calculating the average LWD in three segments of the anterior, mid, and posterior parts of the lungs, with each segment containing the same number of coronal slices with lung tissue in them.

### Statistical analysis

Statistical analysis was performed using GraphPad Prism 9 (GraphPad Software, Inc, La Jolla, California, USA). Continuous variables were reported as mean ± standard deviation (SD). Global and regional LWD, lung volumes, and cardiac output between positions were compared using one-way ANOVA. Comparisons between anterior and posterior LWD within the same image was assessed with student’s t-test. Agreement between measured and known water densities in the phantom were described with intraclass correlation coefficient (ICC) and Bland–Altman analysis. Pearson correlations (R^2^) were reported. Levels of agreement for ICC was defined as poor (0.00–0.30), weak (0.31–0.50), moderate (0.51–0.70), strong (0.71–0.90), and excellent (0.91–1.00) [30]. Threshold for statistical significance was p < 0.05.

## Results

### Phantom validation

Relative water concentration measured in three repetitions by the proton density weighted UTE sequence demonstrated a linear relationship (y = 1.07x − 0.73, R^2^ = 0.98, p < 0.001) with excellent agreement (ICC = 0.97) and low bias (4.3 ± 4.8%) compared to known water concentrations (Fig. 2B, C). The ICC of each repeated experiment was 0.96, 0.99 and 0.98, respectively. This validates that the sequence is proton density weighted, suggesting that the water density quantification from the CMR images is accurate across a range of proton densities, and reproducible.

### Lung segmentation

The U-Net selected for lung segmentation had 3 down sampling layers, 1 branch layer, and 4 up sampling layers, and with a batch size of 64. The trained network had a mean validation loss of 0.096 and a Dice coefficient of 0.93 ± 0.17 in the validation set, indicating good performance compared with the semi-automated method with manual corrections.

**Fig. 5:**
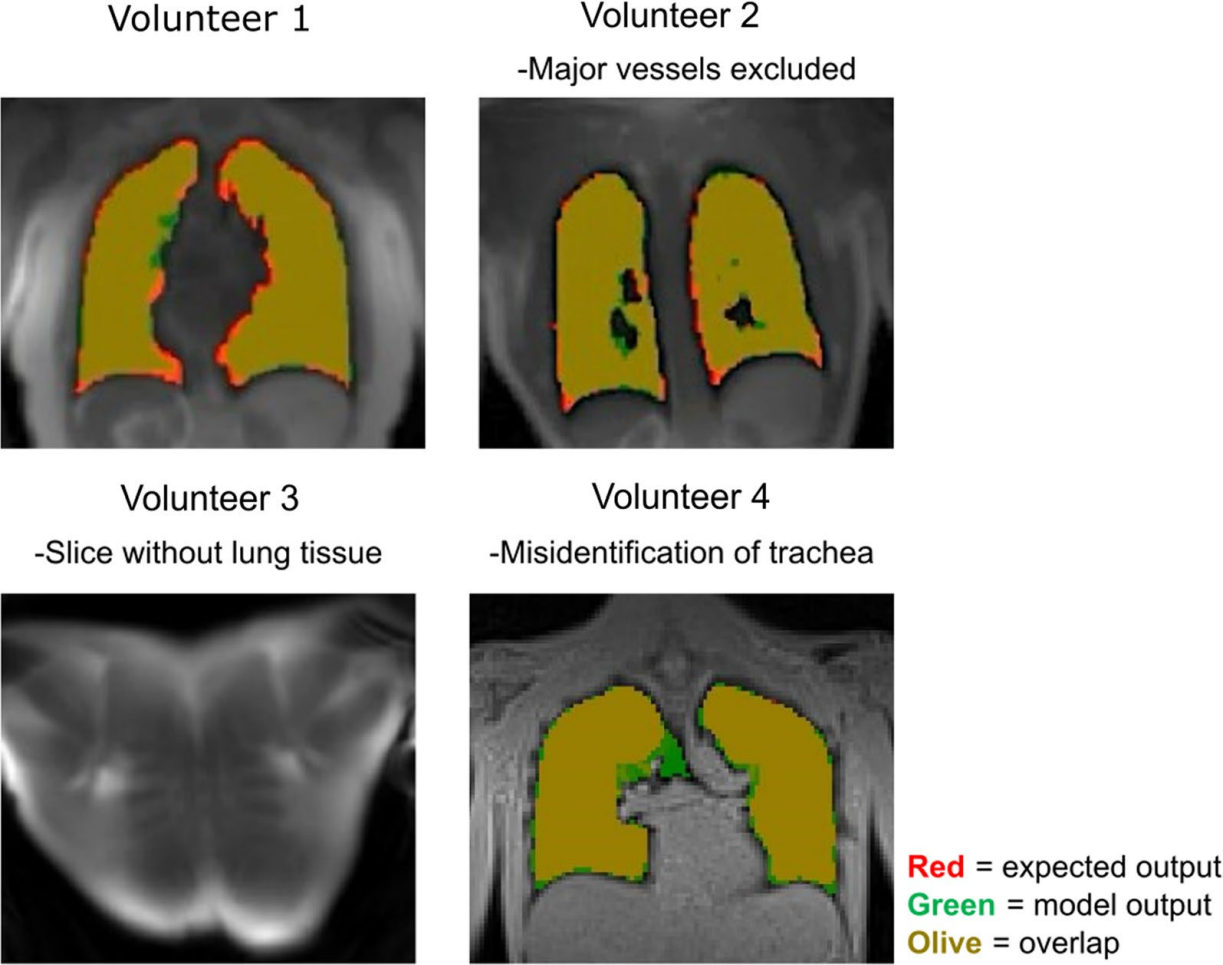
Representative segmentations from the U-Net algorithm from four slices in four healthy subjects. Red shows expected output mask, green shows model output mask, and orange shows the overlap between the two masks. The U-Net created segmentation masks are comparable to the expected output (volunteers 1, 2) and successfully identified image slices where no lung tissue was present (volunteer 3). However, the model mistakenly identified the lower airways as lung in 0.37% of the annotated pixels (volunteer 4)

When evaluated in the separate test dataset of 761 3D images, the final segmentation network had a dice coefficient of 0.93 ± 0.19, indicating comparable performance to the semi-automated segmentation. The average difference in lung volume was − 0.11 ± 0.14 L between the semi-automated and neural network segmentations. The U-Net was able to create accurate masks, with the exclusion of major blood vessels, and successfully exclude image slices where no lung tissue was present (Fig. 5). Average model inference time to segment a 3D lung image with the U-Net was 1.07 ± 0.1 s. Compared to approximately 10 min required by the active contour algorithm with manual corrections, the U-Net demonstrates a significant decrease in processing time. Lung volumes quantified from the U-net segmentations in the first supine images were smaller than in the prone images (2.6 ± 0.9 L vs 2.7 ± 0.9 L, p < 0.001), and there was no difference between the first and second supine images (2.6 ± 0.9 L vs 2.5 ± 1.0, p = 0.29).

### Inline image analysis

Inline calculation of LWD maps was successful in all 15 healthy subjects. The lower airways were partially included in the lung region in all 15 subjects and required offline manual correction in Segment. The misclassified airway pixels amounted to 0.37 ± 0.36% of the total amount of pixels annotated as lung in the automated segmentation. The global LWD using the inline automated processing and manually corrected segmentations did not differ (24.2 ± 3.5% vs 24.5 ± 3.5%, p = 0.16). The liver ROI was accurately positioned in all 15 subjects. Image reconstruction and image analysis required 28.1 ± 2.5 s and 23.2 ± 0.3 s, respectively. The 23 s for image analysis included ~ 1 s of U-net inference and ~ 9 s to stream data between Matlab and Python interfaces, and ~ 13 s for spatial normalization including the L-curve smoothing parameter optimization. The mean optimized L-curve smoothing parameter used for the Tikhonov regularization was 40.8 ± 0.12. This low parameter value variation suggests that the smoothing parameter does not need to be optimized for each image acquired with the same system and sequence parameters, which would reduce the time required for image analysis.

### Lung water density quantification

**Fig. 6:**
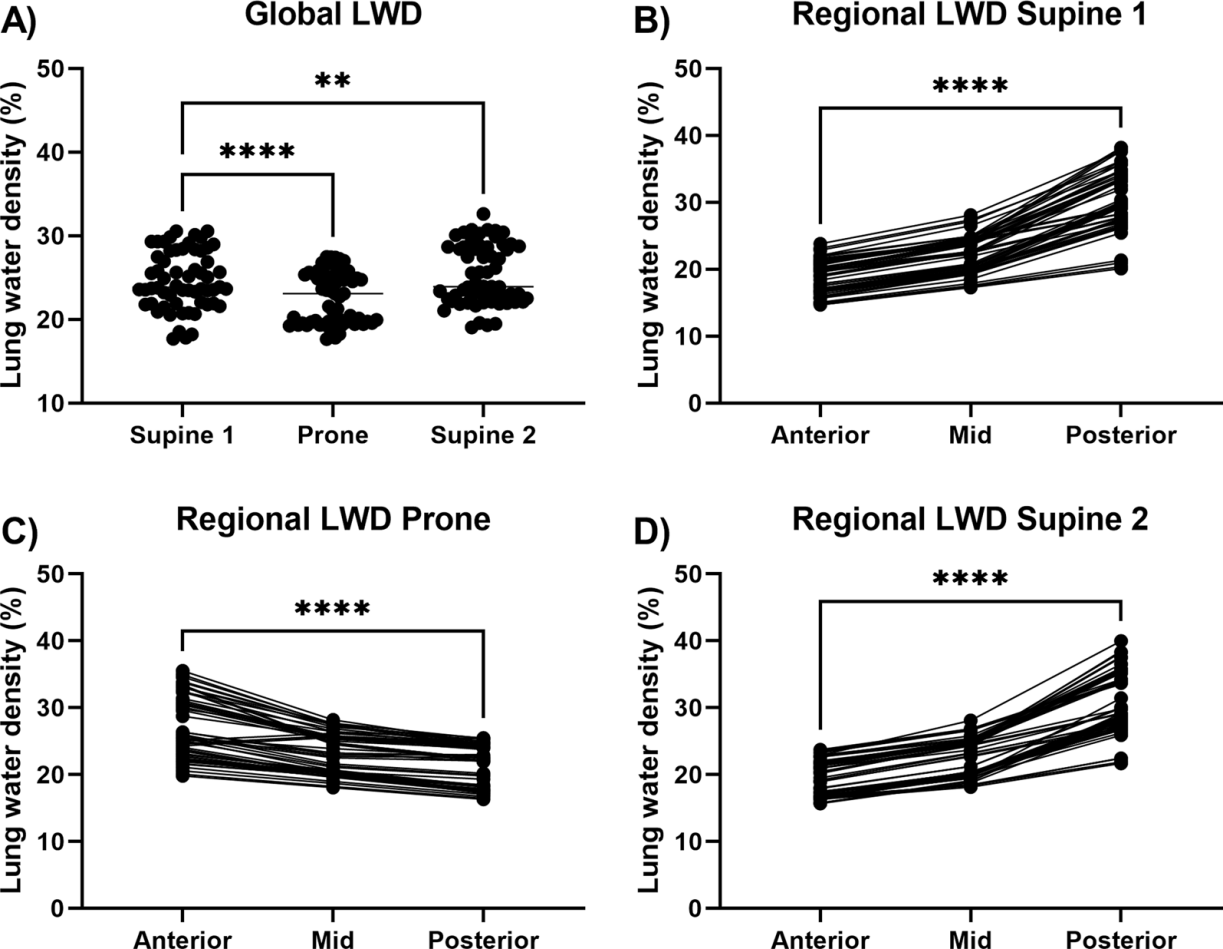
Quantified global lung water density (LWD) in 15 healthy subjects. **A** Global LWD was varied slightly between imaging positions. Regional LWD was higher in the posterior lungs when supine (**B**, **D**) and in the anterior lungs when prone (**C**), indicating that a redistribution of lung water due to gravity can be depicted. **p < 0.01; ****p < 0.0001

The supine and prone imaging protocol was feasible in all included study participants. The average global LWD was comparable between imaging positions (supine 24.7 ± 3.4%, prone 22.7 ± 3.1%, second supine 25.3 ± 3.6%) (Fig. 6A). There were small but significant differences in global LWD between the three imaging phases (first supine vs prone 2.0%, p < 0.001; first supine vs second supine − 0.6%, p = 0.001; prone vs second supine − 2.7%, p < 0.001). The agreement in global LWD between the first and following three acquired images in each position was excellent (ICC = 0.97, bias ± SD − 0.17 ± 0.91%). This demonstrates that the proposed LWD metric is repeatable. Global and regional lung water densities over all phases and repetitions are summarized in Table 1. Global lung water densities were slightly higher in the left lung compared to the right lung (24.0 ± 3.4 vs 24.9 ± 4.3, p < 0.001).

**Table 1:** Quantified lung water densities

	Repetition number	Healthy subjects (n = 15)		
		Phase 1, supine	Phase 1, prone	Phase 3, supine
Global LWD (%)	1	24.3 ± 3.6	22.5 ± 3.2	25.4 ± 4.0
	2	24.7 ± 3.5	22.7 ± 3.3	25.2 ± 3.5
	3	24.8 ± 3.6	22.6 ± 3.1	25.3 ± 3.5
	4	24.9 ± 3.4	22.9 ± 3.2	25.2 ± 3.6
LWD, anterior lungs (%)	1	21.2 ± 3.6	19.7 ± 3.4	19.9 ± 2.9
	2	21.2 ± 3.8	19.8 ± 3.3	19.9 ± 2.8
	3	21.3 ± 3.5	19.8 ± 3.2	20.0 ± 3.1
	4	21.4 ± 3.6	19.8 ± 3.3	20.0 ± 3.3
LWD, mid lungs (%)	1	23.5 ± 3.6	21.8 ± 2.7	22.0 ± 2.4
	2	23.5 ± 3.5	21.9 ± 2.5	22.1 ± 2.3
	3	23.5 ± 3.4	21.9 ± 2.4	22.1 ± 2.5
	4	23.6 ± 3.4	22.0 ± 2.4	22.2 ± 2.7
LWD, posterior lungs (%)	1	28.1 ± 7.8	26.3 ± 4.9	27.2 ± 5.8
	2	28.2 ± 7.2	26.7 ± 5.0	27.3 ± 5.9
	3	28.3 ± 7.6	26.9 ± 5.1	27.2 ± 5.7
	4	28.4 ± 7.5	27.0 ± 5.1	27.2 ± 5.7

Values reported as mean ± standard deviation

*LWD*, lung water density

### Gravity-induced lung water redistribution

**Fig. 7:**
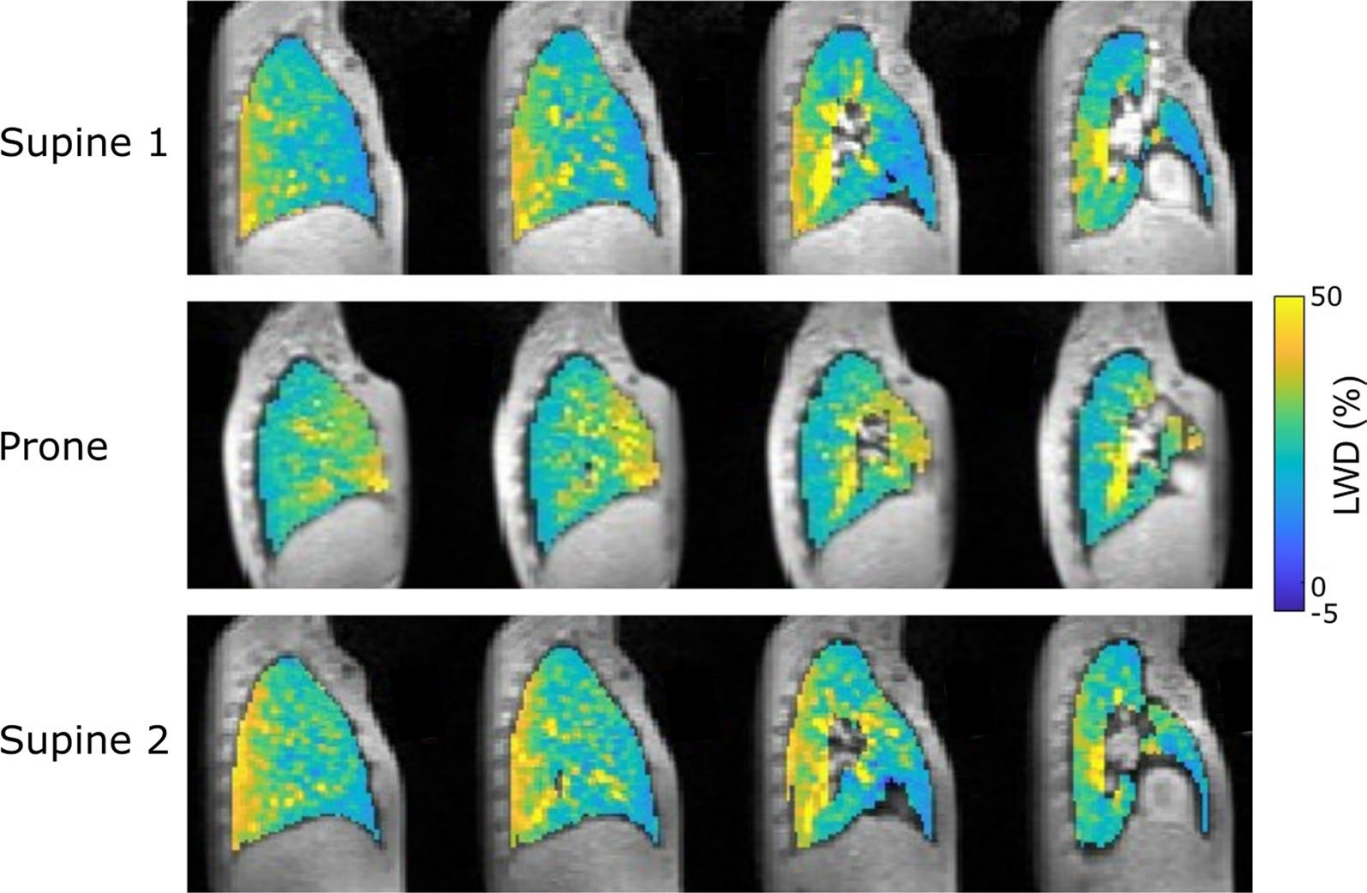
Lung water density (LWD) maps derived from images in the supine and prone positions in four sagittal slices from an example healthy subject. Posterior part of the lung is to the left, and anterior to the right, demonstrating a gravity-induced redistribution of lung water. The LWD was predominantly accumulated in the posterior lungs when supine and in the anterior lungs when prone

We observed a well-defined redistribution of lung water alternating between the posterior parts of the lungs when imaged in a supine position, to the anterior parts when prone (Fig. 7). This change in lung water distribution illustrates the effect gravity has on the pulmonary fluid distribution. The variation between positions was quantified using the difference in LWD between the anterior and posterior lung regions (supine − 11.6 ± 2.7%, p < 0.0001; prone 5.5 ± 2.7%, p < 0.001; second supine − 11.4 ± 2.9%, p < 0.001) (Fig. 6B–D). Repositioning of the subject followed by shimming and localizers took on average 6 min. Lung water redistribution had occurred before the first image in each new position, and no evolution in LWD was observed during four repeated measurements. The variation of the anterior–posterior LWD difference between the first and following three acquired images was small in all imaging positions (supine 0.08 ± 0.7%, prone 0.44 ± 1.0%, second supine − 0.02 ± 0.5%). This suggests that the redistribution of lung water due to gravity occurs more rapidly than the CMR images were acquired in this study. Cardiac output did not differ between imaging positions (supine 6.1 ± 1.0 cm/s; prone 6.2 ± 1.1 cm/s, p = 0.80; second supine 6.2 ± 1.3 cm/s, p = 0.89).

## Discussion

This study presents a method to quantify lung water density in ~ 2.5 min, using a free-breathing stack-of-spirals 3D UTE CMR sequence at 0.55 T with isotropic resolution along with a fully-automated, inline, image-processing pipeline. The method was capable of measuring the redistribution of lung water due to gravity, and can easily be added to a CMR exam. The accuracy and repeatability of the method was validated in a phantom, and the capability to depict a redistribution in LWD was demonstrated by imaging healthy subjects in the supine and prone position. Image processing was performed inline using the Gadgetron framework, with Matlab integrated, and required ~ 23 s after image reconstruction.

### Inline image processing

A streamlined image analysis process is important for widespread clinical adoption of a novel imaging test. Therefore, we proposed an automated, inline image segmentation and lung water quantification that displays results directly on the scanner, enabling on-the-spot physician evaluation without need for additional image analysis, likely using third-party software. Accurate lung segmentation is an important processing step to quantify global and regional LWD, and we implemented and trained a neural network specifically for this task as deep-learning algorithms are robust and reduces processing times. By comparison, previously proposed region-growing algorithms may fail in images with low contrast, or manual segmentations are time-consuming [12, 13]. The neural network performed reliably in the 15 healthy subjects in this study, but partially included the lower airways into the lung segmentation. This misclassification did however not impact the global LWD quantification, as the number of pixels pertaining the airways was negligible (~ 0.37%) and there was no fluid in the airways. This misclassification by the U-net is explained by the anatomical proximity of the lungs and airways, as well as their low signal intensities compared to surrounding musculoskeletal tissues. Moreover, the U-net was mainly trained on images of healthy volunteers and patients with lymphangioleiomyomatosis, and may therefore perform inferiorly on other cohorts with different pulmonary features such as pleural effusion. Retraining the network using more data, including additional patient cohorts, may further improve the robustness and versatility of the segmentations. The neural network provided a fast, accurate, and robust lung segmentation (~ 1 s), which enabled estimation of global LWD in parity with manual image processing. Although inline processing is valuable in the clinical setting, the implementation of the image analysis pipeline into Segment is also powerful as it supports offline analysis that allows for potential manual corrections and is useful in the process of research and development.

### Quantitative lung water imaging

Quantitative proton density weighted UTE imaging requires a stable signal reference with known water density [31]. Thompson et al. have previously proposed hepatic tissue as signal reference, by assuming a 70% water density in the liver [6]. The liver is always within the field-of-view, and it is straight forward to automatically detect within the acquired image after performing lung segmentation. Receiver coil shading correction is another important aspect to achieve quantitative CMR. We performed coil shading as previously proposed by Meadus et al., using a spatial signal fit with Tikhonov regularization directly on the acquired UTE images, as a part of their image post processing [12]. This approach is suitable for inline image processing as it only requires the acquired image itself and is fast. The time to perform spatial normalization could be reduced further if the smoothing parameter was set to a fixed value rather than optimized for each acquired image, but further studies are warranted to determine if this would be suitable over a variety of patient cohorts. Another approach would be to perform coil shading normalization utilizing separate body and surface coil acquisitions, which would provide actual coil signal profiles. The quantified global LWD in healthy volunteers in this study (first supine 24.7%) was comparable with previously reported findings by Meadus et al. using a UTE yarnball sequence at 3 T (28.6%) [12], but higher than findings by Thompson et al. using a HASTE sequence at 1.5 T (16.6%) [6]. Direct comparisons of reported LWD values are however challenging, as results may vary with different sequences, contrast weighting, signal references, and lung segmentation strategies.

### Dynamic lung water CMR

Quantification of lung water dynamics may provide a fast and noninvasive clinical test that can be used to diagnose and monitor patients, and could potentially predict heart failure with preserved ejection fraction in patients who have elevated left ventricular filling pressures and pulmonary congestion. In our study, the changes in lung water were gravity-induced by imaging subjects in the supine and prone positions. This is not expected to be a clinically relevant test for assessment of dynamic changes in LWD, but was rather performed for validation purposes as a way to evaluate the method’s sensitivity to redistribution of lung water. Accordingly, a redistribution was observed upon repositioning each subject, where the predominant LWD accumulation shifted from the posterior parts of lungs in the supine position to the anterior when prone. The low difference in global LWD between positions was expected, as images were acquired under similar physiological conditions, and was further corroborated by consistency in cardiac output in the supine and prone positions. This confirms previous findings by Wieslander et al. [32]. The reason for the small but significant increase of lung water between the first and second supine imaging phases is unknown, and may be attributed to a slight lung water accumulation after laying down for approximately 1 h. The slightly higher lung water density in the left lung compared to the right lung confirms previous findings by Meadus et al., and is explained by its proximity to the heart and major vasculature [12].

A more physiologically relevant assessment of dynamic changes in lung water is during exercise. Lung water measurements during exercise could potentially detect latent heart failure at early stages of the disease where filling pressures and lung water are normal at rest, but markedly increased during exercise. Recently, Burrage et al. [13] imaged heart failure patients and healthy controls directly after exercising for 6 min using a CMR-compatible ergometer. That study demonstrated significant stress-induced increase in lung water of up to 4.4% and 6.4% in patients with heart failure and amyloidosis, respectively. The exercise-induced effects on regional lung water distribution were however not studied in their single 2D axial slice of the lungs, and we anticipate that our proposed 3D self-gated UTE implementation which allows global and regional assessment of LWD and provides stable measurements, may enable a more comprehensive assessment of lung water that is well-suited for application in conjunction with exercise. The lung water reabsorption time after exercise recovery is expected to vary across patient cohorts depending on filling pressures and amount of transudate fluid [33], posing requirements on the acquisition time of dynamic lung water imaging technique during exercise. Agostini et al. have previously reported a decreased alveolar-capillary membrane conductance in heart failure patients at 2 min post exercise but that varies with heart failure severity [33], suggesting that our 2.5 min 3D acquisition might need to be further optimized and shortened in order to observe exercise-induced lung water dynamics. Possible avenues to allow for shorter acquisition times includes increasing the amount of data utilized for the respiratory-binned image reconstruction into a stable phase from an expected rapid respiratory cycle, inclusion of data from the entire respiratory cycle following motion correction [34], or modifying reconstruction for additional undersampling. For acquisition during exercise, we anticipate that the respiratory navigator with temporal resolution of 144 ms will perform well for assessment of rapid respiratory rate and will also detect bulk motion during exercise. Such future studies may enhance the understanding of pathophysiological mechanisms and treatments in patient cohorts presenting with pulmonary edema.

### Low-field strength lung CMR

The proposed image acquisition in our study was developed, optimized, and validated specifically for high-performance 0.55 T CMR. This novel system was created by ramping down a clinical 1.5 T system to maintain hardware performance at a lower field strength [15]. A clinical CMR system operating at lower field strength offers improved B0 field homogeneity (linear with field strength for the same superconducting magnet design), and thus reduced susceptibility gradients at air-tissue interfaces in the lung parenchyma. This system configuration has been demonstrated for high quality structural and functional lung imaging [15–17, 35, 36]. Our optimized stack-of-spirals acquisition exploits the system field homogeneity and prolonged T2* for signal-to-noise-efficient 5.0 ms spiral readouts enabling high-quality isotropic 3.5 mm resolution proton density images within a 2.5 min acquisition [14, 37], with a nominal variation in acquisition time depending on the required amount of slices. Compared to the high-resolution T1-weighed UTE imaging presented in Javed et al. [14], the sequence in this study used a reduced flip angle (from 5° to 1°) for proton density weighting, and a lower spatial resolution (from 1.75 to 3.5 mm) with associated reduced acquisition time (from 15.5 to 2.5 min) by reducing the number of spiral interleaves (from 911 to 171). Additionally, the cardiac diagnostic imaging capabilities are comparable to conventional acquisitions at 1.5 T [15, 38], meaning that this CMR system could be applied for comprehensive functional cardiopulmonary imaging exam [39].

Methods for measuring lung water with CMR have however successfully been developed for 1.5 T and 3 T systems [6, 12, 13]. By comparison, Meadus et al. acquired pulmonary images at 3 T using an efficient 3D yarnball trajectory for their UTE sequence with isotropic 2.5 mm resolution in ~ 2 min [12]. We anticipate that our sequence and image reconstruction and processing pipeline could be translated to 1.5 T and possibly also 3 T following optimization of stack-of-spirals acquisition and retraining the neural network on higher field strength data.

### Limitations

Our study has some notable limitations. First, the proton density weighted sequence does not discriminate between intravascular and extravascular fluid, both of which will redistribute due to gravitational forces. Although the heart and major vessels were excluded from the lung segmentation, it is inevitable that part of the quantified LWD originates from spins in the extensive pulmonary capillary bed. This could potentially have implications in the LWD quantification in patients with pulmonary perfusion deficits. Second, an accurate lung water quantification is dependent on a stable and accurate coil shading correction, as well as a stable signal reference with known water density. The assumption of a 70% hepatic water density might not hold in patient cohorts with fat infiltration or iron overload in the liver. Third, the studied cohort was predominantly young and healthy, and future patient studies and further method development are warranted to determine if CMR quantification of dynamic changes in LWD during exercise can unmask latent heart failure.

## Conclusions

Redistribution of lung water density induced by gravitational forces can be depicted and quantified using a free-breathing 3D isotropic proton density weighted UTE sequence on a high-performance 0.55 T CMR system, along with a fully automated image processing pipeline.

## Data Availability

Source code for image reconstruction and image processing is available open source (https://github.com/NHLBI-MR/lung_water_pipeline). The datasets used during the current study are available from the corresponding author on reasonable request.

## Abbreviations

CMR: Cardiovascular magnetic resonance
CT: Computed tomography
HASTE: Half-Fourier single shot turbo spin echo
ICC: Intraclass correlation coefficient
LWD: Lung water density
PDw: Proton density weighted
ROI: Region of interest
UTE: Ultrashort echo time
